# Franz Büchners Hypoxieforschung aus der Kriegszeit und der Nobelpreis

**DOI:** 10.1007/s00292-022-01086-0

**Published:** 2022-06-09

**Authors:** Elisa Malik, Timo Baumann, Heiner Fangerau, Nils Hansson

**Affiliations:** Institut für Geschichte, Theorie und Ethik der Medizin, Centre for Health and Society, Medizinische Fakultät, Heinrich-Heine-Universität Düsseldorf, Moorenstr. 5, 40225 Düsseldorf, Deutschland

**Keywords:** Militärforschung, Wissenschaftliche Auszeichnungen, Pathologie, Hypoxie-Forschung, Medizin im Nationalsozialismus, Military research, Scientific awards, Pathology, Hypoxia research, Medicine during National Socialism

## Abstract

**Hintergrund:**

Im Zeitraum zwischen 1901 und 1953 wurden insgesamt 5110 Personen für den Nobelpreis in der Kategorie „Physiologie oder Medizin“ nominiert. Dieser Zeitraum umspannt beide Weltkriege und berührt die Frage nach dem Umgang der Nobelkomitees mit deutschen Preiskandidaten.

**Ziel der Arbeit:**

Anhand der Nobelpreisnominierung des deutschen Pathologen Franz Büchner soll dargestellt werden, inwieweit es für die Preisvergabe eine Rolle spielte, wenn auszuzeichnende Forschungsergebnisse teilweise in der Zeit des Nationalsozialismus gewonnen worden waren. Flankiert wird dieser Artikel von einer Übersicht sämtlicher Pathologen aus Deutschland, die in der ersten Hälfte des 20. Jahrhunderts für den Nobelpreis nominiert wurden.

**Material und Methoden:**

Ausgewertet wurden Daten des Nominierungsarchivs in Stockholm sowie Nominierungsbriefe und Gutachten des Nobelkomitees (Nobelarchiv). Die Nominierung Franz Büchners wird als Beispiel näher beleuchtet, weil die Nominatoren ihren Vorschlag mit hier nachverfolgten Publikationen Büchners begründeten, die teils aus der NS-Zeit stammen.

**Ergebnisse und Diskussion:**

Franz Büchner wurde 1963 von drei Müsteraner Professoren nominiert. Beide Bereiche, für die er ausgezeichnet werden sollte, betrafen seine Forschung über den Einfluss von Sauerstoffmangel auf die Funktion und Entwicklung des menschlichen Organismus. Letztlich wurden Büchners Leistungen als nicht nobelpreiswürdig eingestuft. Seine Rolle als Militärforscher während des Nationalsozialismus und das in diesem Zeitraum erworbene Wissen über Hypoxie scheinen keine negative Auswirkung auf das Nobelpreisgutachten gehabt zu haben.

## Abstract (en)

**Background:**

Between 1901 and 1953, a total of 5110 persons were nominated for the Nobel Prize in Physiology or Medicine. This time period spans both world wars and touches on the question of how the Nobel Committees dealt with German prize candidates.

**Purpose:**

The nominations of the German pathologist Franz Büchner for the Nobel Prize will be used to examine the extent to which it played a role in the awarding of the prize if some of the research results to be honoured were obtained during the National Socialist era. The article also presents an overview of all pathologists from Germany who were nominated for the Nobel Prize during the first half of the 20th century.

**Materials and methods:**

Data from the nomination archive in Stockholm as well as nomination letters and expert opinions of the Nobel Committee (Nobel Archive) were analysed. Franz Büchner’s nomination is examined in more detail as an example, because the nominators justified their proposal with Büchner’s publications traced here, that in part originated from the National Socialist era.

**Results and discussion:**

Franz Büchner was nominated by three German professors in 1963. Both areas for which he was to be awarded concerned his research on the influence of oxygen deficiency on the function and development of the human organism. In the end, Büchner’s achievements were deemed not worthy of the Nobel Prize. His role as a military researcher during National Socialism and the knowledge of hypoxia acquired during this period do not seem to have had a negative impact on the Nobel Prize evaluation.

## Einleitung

Seit 1901 wird der Nobelpreis jährlich an diejenigen vergeben, die – so Alfred Nobel in seinem Testament – „im verflossenen Jahr der Menschheit den größten Nutzen geleistet haben“ [1]. Die Auszeichnung verfügt über eine international einzigartige Reputation [2]. Dieser Beitrag reiht sich ein in ein Forschungsprojekt, das über die Laureaten in der Preiskategorie „Physiologie oder Medizin“ hinausgeht und deutsche Pathologen auflistet, die teils zwar mehrfach, zumeist aber vergeblich für den Preis nominiert wurden. Seit 1901 erhielten insgesamt nur 12 Pathologen den Nobelpreis für „Physiologie oder Medizin“ (Tab. 1). Zwischen 1901 und 1953 – für diesen Zeitraum werden im digitalen Nobelarchiv sämtliche Nominierungen mit Kurzbegründung aufgeführt – wurden 15 deutsche Pathologen für den Nobelpreis nominiert, nur einer (Gerhard Domagk 1939) erhielt ihn (Tab. 2).

**Table 1:** 

Jahr	Nobelpreisträger	Offizielle Begründung des Nobelpreiskomitees
1906	Camillo Golgi (1843–1926)	„…in recognition of their work on the structure of the nervous system.“
1906	Santiago Ramón y Cajal (1852–1934)	„…in recognition of their work on the structure of the nervous system.“
1926	Johannes Fibiger (1867–1928)	„…for his discovery of the Spiroptera carcinoma.“
1929	Christiaan Eijkman (1858–1930)	„…for his discovery of the antineuritic vitamin.“
1930	Karl Landsteiner (1868–1943)	„…for his discovery of human blood groups.“
1934	George Hoyt Whipple (1878–1976)	„…for their discoveries concerning liver therapy in cases of anaemia.“
1939	Gerhard Domagk (1895–1964) Einziger Deutscher	„…for the discovery of the antibacterial effects of prontosil.“
1945	Sir Howard Florey (1898–1968)	„…for the discovery of penicillin and its curative effect in various infectious diseases.“
1966	Francis Peyton Rous (1879–1970)	„…for his discovery of tumour-inducing viruses.“
1975	Renato Dulbecco (1914–2012)	„…for their discoveries concerning the interaction between tumour viruses and the genetic material of the cell.“
1980	Baruj Benacerraf (1920–2011)	„…for their discoveries concerning genetically determined structures on the cell surface that regulate immunological reactions.“
2005	John Robin Warren (1937)	„…for their discovery of the bacterium Helicobacter pylori and its role in gastritis and peptic ulcer disease.“

**Table 2:** 

Name	Nominierungen für den Medizin-Nobelpreis
Karl Joseph Eberth (1835–1926)	*2* *×* *für seine Tätigkeit als Bakteriologe*, 1910 by H Schwartze → „…for the discovery of the typhus bacilli“ 1910 by Julius Bernstein → „…for the discovery of the typhus bacill.“
Paul Flechsig (1847–1929)	*2* *×*, 1923 by Hans Held → „…for investigations on the neural conduction paths in the brain and spinal cord.“ 1924 by Hans Curschmann → „…for work on the functions’ cerebral topic.“
Wilhelm Erb (1840–1921)	*2* *×* 1910 by N Ortner → „…for work on electro diagnostics and neuro pathology.“ 1914 by H Ortner → „…for work on electro diagnostics and neuro pathology.“
Félix Marchand (1846–1928)	*9* *×* 1902 by E Küster → „…for work on physiological chemistry.“ 1911 by Emil Kraepelin → „…for work on pathology.“ 1912 by Friedrich Müller → „…for several works.“ 1912 by Emil Kraepelin → „…for several works on pathology.“ 1912 by Hermann Dürck → „…for work on pathology.“ 1918 by Martin Schmidt → „…for work on moles, chorionepithelioma, wound healing and regeneration.“ 1918 by H Tillmanns → „…for work in the field of general pathology and pathological anatomy.“ 1921 by Friedrich von Müller → „…for work on inflammation, developmental disturbances, arteriosclerosis, blood diseases and bronchial astma.“ 1923 by Hermann Dold → „…for work on inflammation and tumors.“
Ludolf von Krehl (1861–1937)	*2* *×* 1917 by M Matthes → „…for internal medicine issues from the biological view of pathophysiology.“ 1932 by E von Romberg → „…for work on pathophysiology.“
Franz Ernst Christian Neumann (1834–1918)	*3* *×* 1908 by Max Askanazy → „…for work on diseases of the blood, and the discovery of the bone marrow’s function in the formation of blood.“ 1909 by P von Baumgarten → „…for the discovery of the bone marrow’s function in the formation of blood.“ 1917 by P von Baumgarten → „…for work on the importance of the bone marrow for the production of blood and in leukemia.“
Rudolf Virchow (1821–1902)	*3* *×* 1901 by Ramon Jimenes Garcia → keine Begründung angegeben. 1901 by José Ribera y San → keine Begründung angegeben. 1902 by R von Jaksch → „…for his importance to modern medicine. Work on leukemia, and in pathology.“
Ludwig Aschoff (1866–1942)	*9* *×* 1917 by M Matthes → keine Begründung angegeben. 1920 by O von Schjerning → „…for work on constitutional and infectious diseases and the normal nature of organs.“ 1923 by Otto Naegeli → „…for clinical pathological studies, work on general pathology and the heart.“ 1930 by N Ortner → „…for numerous research results in pathological anatomy.“ 1930 by A Policard → „…for work on the reticuloendothelial system.“ 1933 by Walther Fischer → „…for work on the reticuloendothelial system.“ 1933 by H Eppinger → „…for work on the reticuloendothelial system.“ 1934 by Otto Naegeli → „…for work on the reticuloendothelial system.“ 1934 by Franz Orsos → „…for work on ateroschlerosis, inflammation.“
Franz Büchner (1895–1991)	/(nach 1953, wird in unserem Beitrag behandelt.)
Robert Rössle (1876–1953)	*3* *×* 1950 by R Siebeck → „…for work on inflammation and allergy.“ 1950 by Viktor Patzelt → „…for work on inflammation and allergy.“ 1951 by Erich Hoffmann → „…for tnflammation, step-wise tumour formation and parallels between foetal and maternal tissues in placenta formation.“
Paul Gravitz (1850–1932)	*1* *×* 1928 by Paul Grawitz → „…for general theory of inflammation“; „induktiv aufgebaute Entzündungstheorie“. Hat sich selbst nominiert
Ernst Leopold Salkowski (1844–1923)	*1* *×* 1907 by E Riegler → keine Begründung angegeben
Martin Benno Schmidt (1863–1949)	*1* *×* 1933 by G Burchard → „…for work on the effect of an iron-deficient diet on the blood and body.“
Gerhard Johannes Paul Domagk (1895–1964)	Nobelpreis 1939 gewonnen: „…for the discovery of the antibacterial effects of prontosil.“ *9* *×* 1938 by George Raiziss → „…for discovery of prontosil.“ 1938 by P Mazé → „…for discovery of prontosil.“ 1939 by A Gardne → „…for discovery of the antibacterial effects of prontosil.“ 1951 by O Grutz → „…for thiocarbazone substances (especially conteben) and their effect on tuberculosis and lepra.“ 1951 by H Eyer → „…for thiocarbazone substances (especially conteben) and their effect on tuberculosis and lepra.“ 1952 by H Loebell → keine Begründung angegeben. 1953 by A Peiper → keine Begründung angegeben. 1953 by Max Bürger → keine Begründung angegeben. 1953 by Heinrich Bredt → keine Begründung angegeben
Max Askanazy (1865–1940) Deutscher, Wirkungsfeld in Deutschland und später Schweiz	*1* *×* 1937 by A Jentzer → „…for work on the hematopoietic apparatus, the endocrine glands, animal parasites, and the etiology of cancer.“
Richard Thoma (1847–1923)	*1* *×* 1901 by Leopold Schrötter → „…for work on arteriosclerosis“

^a^Wissenschaftler, die an einem Institut für Pathologie bzw. pathologische Anatomie tätig waren

Der Zeitraum überspannt beide Weltkriege und verweist auf den Umgang mit deutschen Preiskandidaten. Nach dem Ersten Weltkrieg kritisierten insbesondere die schwedischen Sozialdemokraten das Nobel-Komitee, das Fritz Haber – dem *Vater des Giftgaskrieges* [3] – 1919 einen Nobelpreis verlieh, aber ohne Erfolg. Die Preisjury sah nur, dass Habers Ammoniaksynthese der Menschheit Kunstdünger geschenkt hatte. Die deutsche Presse feierte: „Welch ein deutscher Sieg!“ [4].

Der Nürnberger Ärzteprozess (09.12.1946–19.07.1947) hatte deutlich gemacht, dass die in KZs unter Zwang durchgeführte Forschung systematisch betrieben worden war. In Deutschland fokussierte sich das Bild begangener Verbrechen allerdings in der Folge des Prozesses auf rund 350 Haupttäter; Mitläufer bei den Medizinverbrechen wurden (lange) vergessen [5]. Nach welchen Kriterien aber wurden deutsche Mediziner, die für den Nobelpreis nominiert wurden, nach 1945 in Schweden beurteilt und spielte ein möglicher Bezug der Forschung zu Medizinverbrechen im NS eine Rolle in der Diskussion? Am Beispiel einer Serie von Vorschlägen, die im Jahr 1963 alle Franz Büchner nannten, sollen diese Fragen diskutiert werden.

## Franz Büchner: Eine biografische Skizze

Franz Büchner wurde am 20. Januar 1895 in Boppard am Rhein geboren. 1917 begann er das Medizinstudium an der Universität Münster mit Studienortwechseln nach Heidelberg 1918 und Gießen 1921, wo er schließlich sein medizinisches Staatsexamen ablegte. Von 1922 bis 1933 arbeitete er als Assistent Ludwig Aschoffs am Pathologischen Institut der Freiburger Universität [6]. Unter dessen Leitung habilitierte sich Büchner 1927 mit der Arbeit „Die Histologie der peptischen Veränderungen und ihrer Beziehungen zum Magenkarzinom“ [7]. Bereits als Assistent befasste sich Büchner mit den Erkrankungen des Magens und des Magen-Darm-Traktes sowie mit der Pathologie der Koronargefäße, der Angina pectoris und der Herzinsuffizienz. 1933 zog es ihn an das Pathologische Institut des Berliner Krankenhauses am Friedrichshain, bevor er 1936 den Lehrstuhl für Allgemeine und Spezielle Pathologie der Universität in Freiburg von Ludwig Aschoff übernahm. Zu Beginn seiner Freiburger Zeit trug Büchner zur Interpretation und Deutung des Elektrokardiogramms bei Durchblutungsstörungen des Herzmyokards bei [8]. Seit April 1940 leitete er zudem das „Institut für luftfahrtmedizinische Pathologie“ in Freiburg i. Br. als Außenstelle des Reichsluftfahrtministeriums. Seither fokussierte Büchner seine Forschung auf die Hypoxie und ihre Auswirkungen auf Organsysteme sowie später auf die Embryonalentwicklung.

## Büchners Nominierungen für den Nobelpreis

Im Januar 1963 reichte der Direktor des Instituts für Geschichte der Medizin an der Universität Münster, Karl Eduard Rothschuh, eine Nominierung für Franz Büchner beim Nobelkomitee ein. Rothschuh war mittlerweile zwar Medizinhistoriker, aber von 1937 bis 1957 in der Münsteraner Physiologie tätig gewesen. Als Begründung nannte er Büchners „Entdeckung, dass Sauerstoffmangel 1) strukturzerstörend im Herzen, im Gehirn, in der Leber wirkt, 2) in bestimmten Stadien der Embryonalentwicklung nicht nur Missbildungen erzeugen kann, sondern dass der O_2_-Mangel je nach dem Zeitpunkt der Einwirkung verschiedene Bildungsprozesse bevorzugt zerstört.“ Büchners Leistung liege darin, im Tierversuch nachgewiesen zu haben, dass Mangeldurchblutung, CO-Vergiftung und Sauerstoffmangel Parenchymnekrosen hervorriefen. Rothschuhs Kurzzitat zum Sauerstoffmangel („1937“) bezeichnet sehr wahrscheinlich Büchners Artikel über „die pathogenetische Bedeutung der Hypoxämie“ (Jahrbuch Nobelarchiv: Rothschuh 21.01.1963:12–14). Büchner schrieb darin u. a., die „Übertragung der durch Höhenphysiologie und Luftfahrtmedizin gewonnenen Beobachtungen auf die allgemeine Pathologie wäre noch nicht möglich gewesen, wenn nicht die moderne Physiologie uns wichtige neue Erkenntnisse über die normale Kreislaufarbeit vermittelt hätte“ [9].

Die von Rothschuh aufgezählten Bereiche standen in Verbindung mit Themen, an denen Büchner insbesondere während des Zweiten Weltkriegs geforscht hatte. Im Laufe des Zweiten Weltkrieges hatte Büchners Luftwaffeninstitut immer enger mit dem Luftfahrtmedizinischen Forschungsinstitut des Reichsluftfahrtministeriums in Berlin zusammengearbeitet. Mitte 1944 schrieb dessen Leiter, der Physiologe Hubertus Strughold, der von ihm selbst in die Debatte eingebrachte Begriff „Hypoxydose“ umfasse die „*Störung* der energieliefernden Reaktionen“ durch Sauerstoffmangel in der Zelle als Folge von Hypoxämie, dem Sauerstoffmangel im Blut. Die, so Strughold damals weiter, von Büchner „beschriebene Zellverfettung, Degeneration und Zellnekrosen“ seien „zu betrachten als morphologischer Ausdruck der gestörten oxydativen Prozesse, also einer Hypoxydose.“ Strughold bezog sich auf einen Beitrag Büchners in der *Zeitschrift für Luftfahrtmedizin* von Juli 1942 [10].

Büchner betonte darin, nicht die Hypoxämie, sondern die von ihr entsprechend der Klassifikation durch Strughold ausgelöste Hypoxydose sei bei der „Höhenkrankheit“ entscheidend. Versuche an Katzen wären nicht ausreichend, weil sie so „unterdruckfest“ seien, dass darüber nicht sicher auf das zentrale Nervensystem des Menschen im Sauerstoffmangel geschlossen werden könne. Er forderte in dem im Juni 1942 eingegangenen Aufsatz, die „tatsächlichen Verhältnisse am Menschen exakt zu prüfen“ [11]. Eine vergleichbare Aussage machte er später nicht mehr.

Im Oktober desselben Jahres führte die Sanitätsinspektion der Luftwaffe in Nürnberg eine „Besprechung“ über „ärztliche Fragen bei Seenot und Winternot“ durch. Unter 95 Teilnehmern trug Franz Büchner über seine „histologische Durcharbeitung von 20 Unterkühlungstodesfällen“ vor [12]. In einem anderen Vortrag führte Ernst Holzlöhner aus, dass in kalter See verunglückte Menschen nach der Bergung oft plötzlich verstarben („Rettungskollaps“). Dies werde im Tierversuch „niemals“ beobachtet. Holzlöhner hatte „Menschen, die nach längerem Aufenthalt in kaltem Wasser geborgen wurden“, untersucht. Bewirke die Kälte eine Körpertemperatur von 29° oder 30°, dann stelle sich Vorhofflimmern ein, das wieder verschwinde. „Hat die Rektaltemperatur aber 28° unterschritten, so kann aus der Arrhyt[h]mie heraus ein plötzlicher Herztod erfolgen“ [13]. Dem nur für den Dienstgebrauch gedruckten Protokoll ist nicht direkt zu entnehmen, dass Holzlöhner im Konzentrationslager Dachau für Forschungen, die er für Luftwaffe und SS durchführte, Experimente an Häftlingen durchgeführt hatte, die für einen erheblichen Teil absehbar tödlich verlaufen waren [14].

Alexander Mitscherlich hatte nach dem Krieg in seiner Dokumentation über den Nürnberger Ärzteprozess behauptet, dass kein Teilnehmer während der „Seenot“-Besprechung protestiert habe. Er nannte Büchners Namen dabei aber nicht, sondern erwähnte ihn viele Seiten davon entfernt in einem ganz anderen Zusammenhang: Der Hygieniker Eugen Haagen hatte Mitte 1944 laut einem in der Dokumentation abgedruckten Schreiben einerseits im Umfeld von Hepatitis die Vokabel „Humanversuche“ fallen lassen und andererseits auf seine Zusammenarbeit mit Büchner und einem weiteren Forscher hingewiesen: „Besonders mit Herrn Kalk habe ich natürlich bereits vereinbart, dass wir mit unserem Material derartige Versuche anstellen werden“ [15]. Dies kann zumindest so verstanden werden, dass Haagen Kalk für einen Mann hielt, den er leicht auf Menschenversuche ansprechen konnte, Büchner dagegen nicht. Der hatte sich bereits im November 1941 in einem Vortrag („Der Eid des Hippokrates“) vor fast Tausend Zuhörern gegen den Krankenmord ausgesprochen [16]; und der Dekan der Medizinischen Fakultät an der Universität Berlin, Paul Rostock, schrieb Ende 1942, er sei sogar schon vor diesem Vortrag von „politischen Beanstandungen“ gegen Büchner unterrichtet gewesen [17].

Der Internist Heinz Kalk nahm Proben am Menschen. Er führte im Mai 1944 auf einer Tagung der Beratenden Ärzte in Hohenlychen aus: „Mit der gezielten Leberpunktion, bei der unter Vornahme einer Laparoskopie (Bauchspiegelung) die Punktionsnadel mit dem Auge durch das Laparoskop dirigiert wird (Kalk), wurden 255 Leberpunktionen bei Hepatitis epidemica vorgenommen“ [18]. Bereits Anfang 1947 erklärte Büchner eidesstattlich, im Krieg aus Tierversuchen stammende Leberpräparate auf Bitte von Haagen untersucht zu haben [19]. Büchner begann zumindest später eine Zusammenarbeit mit Kalk, denn im November 1947 erschien von beiden ein gemeinsames Papier darüber, wie „am lebenden Menschen Teile der Leber zur mikroskopischen Untersuchung zu entnehmen“ seien. Die beiden Forscher berichteten, sie hätten 265 Laparoskopien bei Hepatitis epidemica vorgenommen, korrigierten aber noch: „Die Zahl der ohne Zwischenfälle durchgeführten gezielten Leberpunktionen ist inzwischen auf über 600 gestiegen“ [20]. Zum Nachweis, moralisch integer zu sein, publizierte Büchner nach dem Krieg zudem den einstigen Freiburger Vortrag mit seiner Kritik am Krankenmord in Buchform [21].

Was Büchner wissenschaftlich vorhatte, schilderte er in der 1949 gedruckten Fassung seines 1944 auf der Jahresversammlung der Pathologen gehaltenen zentralen Referats über Sauerstoffmangel als Krankheitsursache. Dem „Kollaps jeden Ursprungs“, daneben „vielfach“ der serösen Hepatitis, liege Sauerstoffmangel zugrunde; Büchner wollte ein ganzes Bündel von Erkrankungen einheitlich darauf zurückführen: „Irreversibel“ schädige Sauerstoffmangel die „sauerstoffhungrige Parenchymzelle, also im Herzmuskel die Muskelfaser, in der Leber die Epithelzelle, im Gehirn die Ganglienzelle, an zweiter Stelle die Gliazelle“ [22]. Damit hatte Büchner ein Konzept vorgestellt, mit der er die in die Krise [23] geratene Theoretische Medizin aufzufangen hoffte. Er entwickelte seine breitangelegte Stoffwechseltheorie ausgehend von empirisch gewonnenem Wissen um ein einheitliches Prinzip von Transport- und Umsetzungsstörungen in mehreren Körperorganen. Dies basierte auf Forschungen, die Pathologen und Physiologen besonders in der Zeit des Nationalsozialismus durchgeführt hatten. Büchner selbst wollte mit seiner Forschung – wie er im November 1943 an den oben erwähnten Paul Rostock, nun frischgebackener Leiter des Amtes für medizinische Wissenschaft, gleich schrieb – „durch die grundsätzliche Erforschung der Pathologie des allgemeinen Sauerstoffmangels und ebenso der Unterkühlung und auch durch unsere Untersuchungen der Hepatitis epidemica [Leberentzündung bei Hepatitis A] sowohl der Kriegspathologie wie der Friedenspathologie dienen“ [17].

Nach 1945 wollte Büchner am Forschungsstrang der Hypoxie unbedingt weiterarbeiten. Was seine Teilnahme an der Besprechung *Seenot und Winternot* anging, so betonte er in seiner Autobiografie, er habe sich beim ranghöchsten anwesenden Luftwaffenoffizier im „Foyer“ nach dem Vortrag eines SS-Arztes gegen die hier vorgestellten Menschenversuche verwahrt [24]. Büchner behauptete also nicht, es sei ihm unverständlich geblieben, dass über letale Menschenversuche vorgetragen worden war. Doch als sein Name in der aufsehenerregenden Dokumentation Alexander Mitscherlichs über den Nürnberger Ärzteprozess 1947 fiel, beauftragte Büchner Rechtsanwälte, dagegen vorzugehen [19]. Er verblüffte schon seine Zeitgenossen mit dieser hochfahrenden Reaktion auf Alexander Mitscherlichs Buch: Büchner selbst machte erst mit seinem lautstarken Protest darauf aufmerksam, dass er unter den 95 Teilnehmern der „Seenot“-Besprechung war [25, 601 f]. Vielleicht fühlte er sich wirklich ungerechtfertigt angegriffen. Namentlich genannt worden war er in der Dokumentation wegen Forschungen, die er im Umfeld seines Langzeitprojekts vorantrieb: der Erklärung eines großen Bereichs der Krankheitslehre auf der Basis eines einheitlichen Stoffwechselprinzips.

Diesen Ansatz der Stoffwechselpathologie würdigte 1963 eine weitere Münsteraner Nominierung an das Nobelpreiskomitee vom Pharmakologen Arnold Loeser – der 1944 von Freiburg nach Münster gewechselt war: „Herr Büchner *entdeckte* die Hypoxaemie bzw. Hypoxie als pathogenetisches Prinzip.“ Büchner habe herausgefunden, dass „ein Missverhältnis zwischen Blutangebot und Blutbedarf des Herzmuskels zur Entwicklung von Herzmuskelnekrosen“ führte, und dass „ein solcher kausaler Zusammenhang von Sauerstoffmangel und Organveränderung auch in anderen Organen (Zell- und Gewebsnekrosen in Leber, Gehirn und Gefässsystem) bzw. bei anderen Erkrankungen nachweisbar ist (Kohlenoxydvergiftung, Höhenkrankheit, Schock und Kollaps).“ Weiter habe Büchner „die Bedeutung des Sauerstoffmangels für Entwicklungsstörungen des Keims“ erkannt (Jahrbuch Nobelarchiv: Loeser 25.01.1963).

Auf diesen zweiten Bereich hatte auch Rothschuh hingewiesen: Daran habe Büchner „in der Zeit nach dem Zweiten Weltkrieg (1946 bis heute)“ geforscht. Rothschuhs ältester Beleg dazu ist bekannt, da die von ihm beigelegten Arbeiten Büchners aufgelistet sind (Jahrbuch Nobelarchiv: Rotschuh 21.01.1963): Büchners Freiburger Pathologenteam publizierte 1946, ihm sei es gelungen, an den Eiern von Tritonen (Molchlaich) durch „allgemeinen Sauerstoffmangel in der Unterdruckkammer Missbildungen zu erzeugen.“ Dabei „wurde nicht selten eine Hemmung und Störung der gesamten Hirnentwicklung beobachtet.“ Nahe liege, dass „auch menschliche Missbildungen nicht selten durch allgemeinen Sauerstoffmangel“ entstünden [26]. Loeser begründete seinen Vorschlag mit der Relevanz von Büchners Arbeit, die seiner Meinung nach deutlich über die Grenzen der Pathologie hinausging: „Es ist nur noch darauf hinzuweisen, dass erst nach jahrelanger Auseinandersetzung mit der Büchnerschen Entdeckung die ausserordentliche Tragweite in theoretischer wie klinischer Hinsicht offenbar werden konnte, und dass sie sich für nahezu jedes medizinische – und auch biologische – Arbeitsgebiet als fruchtbar erwiesen hat“ (Jahrbuch Nobelarchiv: Loeser 25.01.1963).

Eine dritte Nominierung, die 1963 Büchner vorschlug, führte ebenfalls die beiden Bereiche an – und ebenfalls keine weiteren. Sie stammte von Willy Giese, dem Pathologen an der Universität Münster. Er und Büchner hatten im Krieg als Pathologen gedient; im Mai 1944 besuchten beide die erwähnte Tagung der Beratenden Ärzte in Hohenlychen [27]. Gestützt auf Büchners Forschungen sowie spätere Erweiterungen anderer Wissenschaftler resümierte Giese: Die durch Sauerstoffmangel bedingten Fehlbildungen am Embryo „gleichen im Endzustand völlig den vererbbaren genetisch bedingten Missbildungen“ (Jahrbuch Nobelarchiv: Giese 22.01.1963).

Alle drei genannten Vorschlagsschreiben aus Münster geben explizit an, dass Büchner erst nach Kriegsende über Sauerstoffmangel als Ursache für Fehlbildungen geforscht habe. Angeborene und dennoch nicht vererbliche Erkrankungen interessierten Pathologen allerdings bereits in der Zeit des Nationalsozialismus. Es ging etwa um Gliedmaßenabschnürungen während der Schwangerschaft infolge von Fäden, die sich aus der Fruchtblase ablösen können: Der Göttinger Pathologe Georg B. Gruber hatte auf der Jahrestagung der Pathologen 1938 dazu ausgeführt, es sei „aus Gründen erbgesundheitlicher Begutachtung nötig, Klarheit über Eigenart und Grenzen amniotischer Fruchtschäden zu erlangen“ [28] – offenkundig also mit Bezug zum „Gesetz zur Verhütung erbkranken Nachwuchses“, das folglich nicht alle Menschen mit angeborenen Erkrankungen betraf [29]. Auch das Anliegen, die Erforschung angeborener Erkrankungen nicht allein den Genetikern zu überlassen, verfolgte Büchner nach dem Ende des Nationalsozialismus anscheinend weiter.

Büchner gehörte vergleichsweise wenig nationalsozialistischen Verbänden an [30]. Schwerster Verbrechen hatte er sich anscheinend nicht schuldig gemacht und vor und nach 1945 sogar Kritik am Krankenmord geübt – mit dem er als Militärforscher allerdings wenig zu tun hatte. Sein Auftritt in der Öffentlichkeit nach dem Krieg zielte offenkundig darauf ab, den Forschungsansatz fortsetzen zu können, Organerkrankungen auf den Stoffwechsel zurückzuführen.

## Diskussion: Nach welchen Kriterien wurde Büchner beurteilt?

Nach den Nominierungen für Büchner im Jahr 1963 wurde Nils Ringertz, Pathologe am Karolinska Institut, vom Nobelkomitee aufgefordert, eine Stellungnahme zu den Nominierungen abzugeben. In dem zweiseitigen Schreiben auf Schwedisch hob Ringertz einleitend hervor, dass Büchner „einer der führenden Pathologen Deutschlands“ sei, der „eine umfassende Publikationsliste einschließlich Lehrbücher vorzuweisen“ habe. Ringertz stufte die Hypoxie als ein Hauptthema Büchners ein, mit dem der sich seit den 1930er-Jahren „alleine und in Kooperation mit jüngeren Mitarbeitern“ befasst habe. Diese Forschungen, so Ringertz, hätten eine große Bedeutung für die klinische Arbeit zu Themen wie Koronarinsuffizienz, Herzinfarkt, Angina pectoris und auch für die elektrokardiografische Analyse von Myokardschäden gehabt. Die Publikationen in den 1940er-Jahren über anoxiebedingte (er meinte vermutlich hypoxiebedingte) Missbildungen am Huhn seien zwar beeindruckend, aber ähnliche Gedanken waren laut Ringertz bereits von anderen Autoren publiziert worden („Schulze 1898, Stockard 1921, Becker 1938“). Abschließend schrieb Ringertz, dass die Arbeiten „der Büchnerschen Schule“ über Sauerstoffmangel ursprünglich zwar bedeutend gewesen seien, „aber heutzutage weiß man, dass auch andere Aspekte neben der Anoxie wesentliche Rollen spielen“. Deshalb, so urteilte Ringertz, „liegt meines Erachtens bei Büchner keine so bedeutende Entdeckung vor, dass er in die engere Auswahl als Nobelpreiskandidat kommen sollte“ (Jahrbuch Nobelarchiv: Ringertz 09.04.1963). Dieses Urteil beruhte also auf der Tatsache, dass Büchner im internationalen Wettbewerb für bedeutende, spezifische Einzelentdeckungen stand, die Ringertz aber als nicht (Nobel-)preiswürdig eingestufte. Franz Büchners Rolle als Militärforscher im Nationalsozialismus wird im Gutachten nicht thematisiert. Ob Sie im Nobelkomitee intern diskutiert wurden, ist bisher unbekannt.

Die Ergebnisse der Forschung Büchners zur Hypoxie wurden anhand von Erkenntnissen im Tiermodel erworben und als solche publiziert. Büchner forderte Mitte 1942, die „tatsächlichen Verhältnisse am Menschen exakt zu prüfen“, nachdem die Unterdruckversuche in Dachau längst begonnen hatten [30].

Auch wenn Büchners Arbeit nicht als *nobelwürdig* galt, spiegelt sich seine Reputation in anderen Auszeichnungen nach der Nominierung von 1963 wider. 1971 erhielt er die Paracelsus-Medaille der deutschen Ärzteschaft, 1972 die Carl-Ludwig-Ehrenmedaille der Deutschen Gesellschaft für Kardiologie und 1981 die Rudolf-Virchow-Medaille der Deutschen Gesellschaft für Pathologie. 1990 wurde ihm das Große Verdienstkreuz der Bundesrepublik Deutschland mit Stern verliehen. Er verstarb am 9. März 1991 in Freiburg im Breisgau.
